# Gallbladder Cancer Manifesting As Cutaneous Metastases: An Uncommon Clinical Scenario

**DOI:** 10.7759/cureus.78515

**Published:** 2025-02-04

**Authors:** Vanisha Pundir, Itish Patnaik, Rohit Gupta, Bhartendu Bhartendu, Tanmay Jain

**Affiliations:** 1 Gastroenterology, All India Institute of Medical Sciences, Rishikesh, IND; 2 Gastroenterology and Hepatology, All India Institute of Medical Sciences, Rishikesh, IND; 3 Pathology and Lab Medicine, All India Institute of Medical Sciences, Rishikesh, IND

**Keywords:** carcinoma gallbladder, cutaneous metastases, direct extension, extra-abdominal metastases, zosteriform dermatoses

## Abstract

Cutaneous metastasis from gallbladder carcinoma is exceptionally rare. Gallbladder carcinoma typically spreads to the liver and lymph nodes, with extra-abdominal metastasis being uncommon. Extra-abdominal metastases may involve sites such as the lungs or CNS. Skin is an infrequent site of metastases. These cutaneous lesions are often misdiagnosed, especially when they precede the diagnosis of malignancy.

We report a rare case of gallbladder carcinoma presenting with cutaneous metastasis in an elderly male. Initially misdiagnosed and treated as a dermatological condition, this case highlights the diagnostic challenges posed by the rarity and diverse clinical manifestations of cutaneous metastases in gallbladder cancer. Limited awareness among clinicians often leads to delayed recognition and treatment. A high index of suspicion is essential for timely diagnosis, enabling early intervention that may help mitigate systemic disease progression and improve patient outcomes.

## Introduction

Cutaneous metastases, while uncommon in internal malignancies, occur in 0.7-9% of cases, most often originating from the lung, colon, or stomach [1,2]. Primary gallbladder carcinoma, a disease that predominantly affects females, infrequently results in skin metastases. This malignancy typically spreads through direct extension, impacting the liver in 60-90% of cases or spreading to lymph nodes. Although extra-abdominal metastases are rare, they may involve sites such as the lung, central nervous system, or orbits [3]. Extra-abdominal metastasis is disseminated through vascular dispersion and tumor cell homing. Cutaneous metastasis of primary gallbladder cancer is sporadic and has an incidence of 0.7-0.9% [4]. This study presents a rare instance of cutaneous metastasis from gallbladder cancer in an elderly man, detailing its evaluation and management while emphasizing the diagnostic challenges encountered in such cases.

## Case presentation

A 69-year-old gentleman, a chronic smoker with no notable medical or significant family history, presented with painless nodular skin lesions on his left arm near the axilla for six months duration. The lesions were not associated with redness, ulceration, itching, fever, or discharge. The patient developed a new lesion in his right upper abdomen after four months along with zosteriform-like lesions in his left axilla and upper back. He was initially treated for a dermatological disorder with antiviral therapy and topical agents.

**Figure 1:**
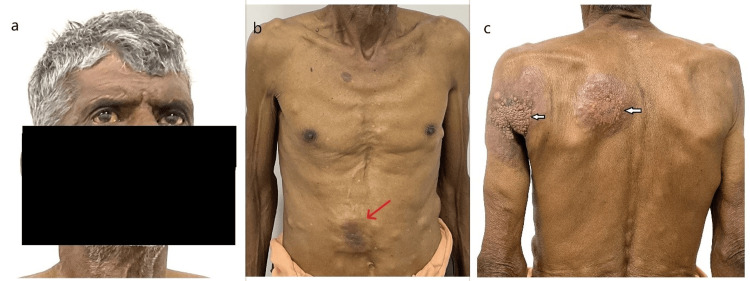
Icterus alongside multiple painless, firm subcutaneous nodules of varying sizes on the chest and abdomen (a-c). The largest lesion, measuring 6x4 cm, is mainly located in the right hypochondrium and extends to the umbilical region (red arrow). Additionally, zosteriform-like lesions (bold white arrows) are observed in the posterior aspect of the left axilla and upper chest.

During the initial assessment, the patient presented with dull epigastric and right upper abdominal pain and yellowish discoloration of the eyes and sclera for one month. Examination findings included pallor and icterus. Multiple, firm, painless nodular subcutaneous deposits of varying sizes were observed on the dorsal and ventral surfaces of the patient's chest and abdomen. The largest of these lesions measured 6x4 cm, located in the right hypochondrium and extending to the umbilical region. This lesion had restricted mobility without adherence to the overlying skin. These nodules were more prominent during the leg-raising test. Additionally, the patient exhibited zosteriform-like lesions on the posterior aspect of the left axilla and left upper chest (Figures 1a-1c). No palpable intra-abdominal mass was detected in the right hypochondrium.

The hematological evaluation revealed hemoglobin of 7.9 g/dL, elevated total bilirubin (10 mg/dL) and direct bilirubin (6 mg/dL), elevated aspartate aminotransferase (AST) (115 IU/L), alanine aminotransferase (ALT) (112 IU/L), alkaline phosphatase (399.8 IU/L), and carcinoembryonic antigen (470 ng/mL).

**Figure 2:**
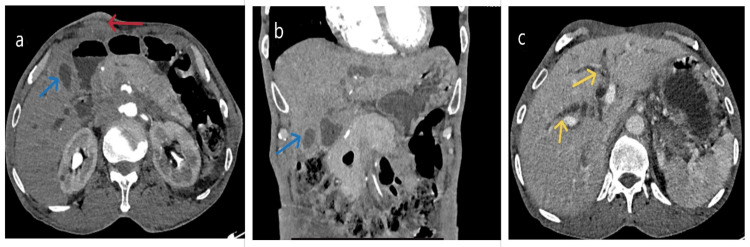
CECT abdomen axial and coronal sections (a-c). Ill-defined, circumferential thickening of the gallbladder's fundus, body, and neck (blue arrow), abutting liver segments IVB, V, and the hepatic flexure of the colon, with indistinct fat planes and associated intrahepatic biliary radicle dilatation (yellow arrows). An enhancing lesion in the skin and subcutaneous tissue of the right hypochondrium (red arrow) which is non-contiguous with the GB thickening. CECT: contrast-enhanced computed tomography; GB: gallbladder

Contrast-enhanced computed tomography (CECT) imaging of the thorax and abdomen revealed an ill-defined, circumferential thickening affecting the gallbladder's fundus, body, and neck, with extension to the proximal common bile duct, resulting in luminal narrowing. The mid-common bile duct narrowing led to upstream dilatation of the intrahepatic biliary radicle. Additionally, a 3.6x1.8x6 cm enhancing lesion was observed within the skin and subcutaneous tissue of the right hypochondrium, suggestive of metastatic deposit. Furthermore, multiple enlarged lymph nodes were noted in the abdominal and mediastinal regions (Figures 2a-2c).

**Figure 3:**
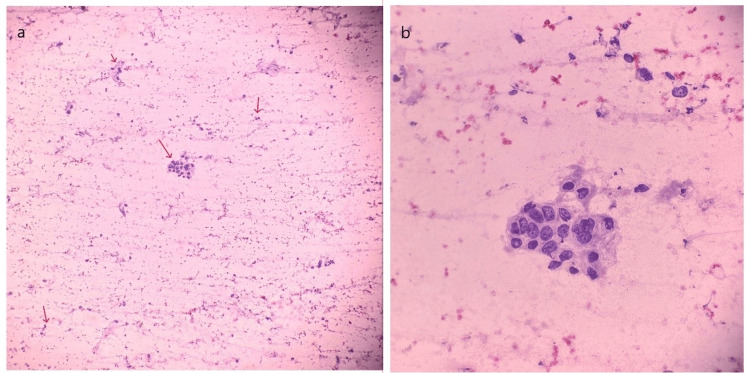
Papanicolaou (PAP) stain. (a) Smear displays singularly lying and small clusters of atypical cells (10x) (red arrows). (b) Image reveals atypical cells with a high nuclear-to-cytoplasmic ratio, enlarged nuclei, moderately pleomorphic chromatin, 1-2 conspicuous nucleoli, and scant to mild cytoplasm suggestive of metastatic adenocarcinoma (40x).

Fine-needle aspiration cytology of the cutaneous lesion revealed clusters of atypical cells with a high nuclear-to-cytoplasmic ratio, enlarged pleomorphic nuclei with vesicular to coarse granular chromatin, and an irregular nuclear membrane. These findings were consistent with an adenocarcinoma (Figures 3a, 3b).

**Figure 4:**
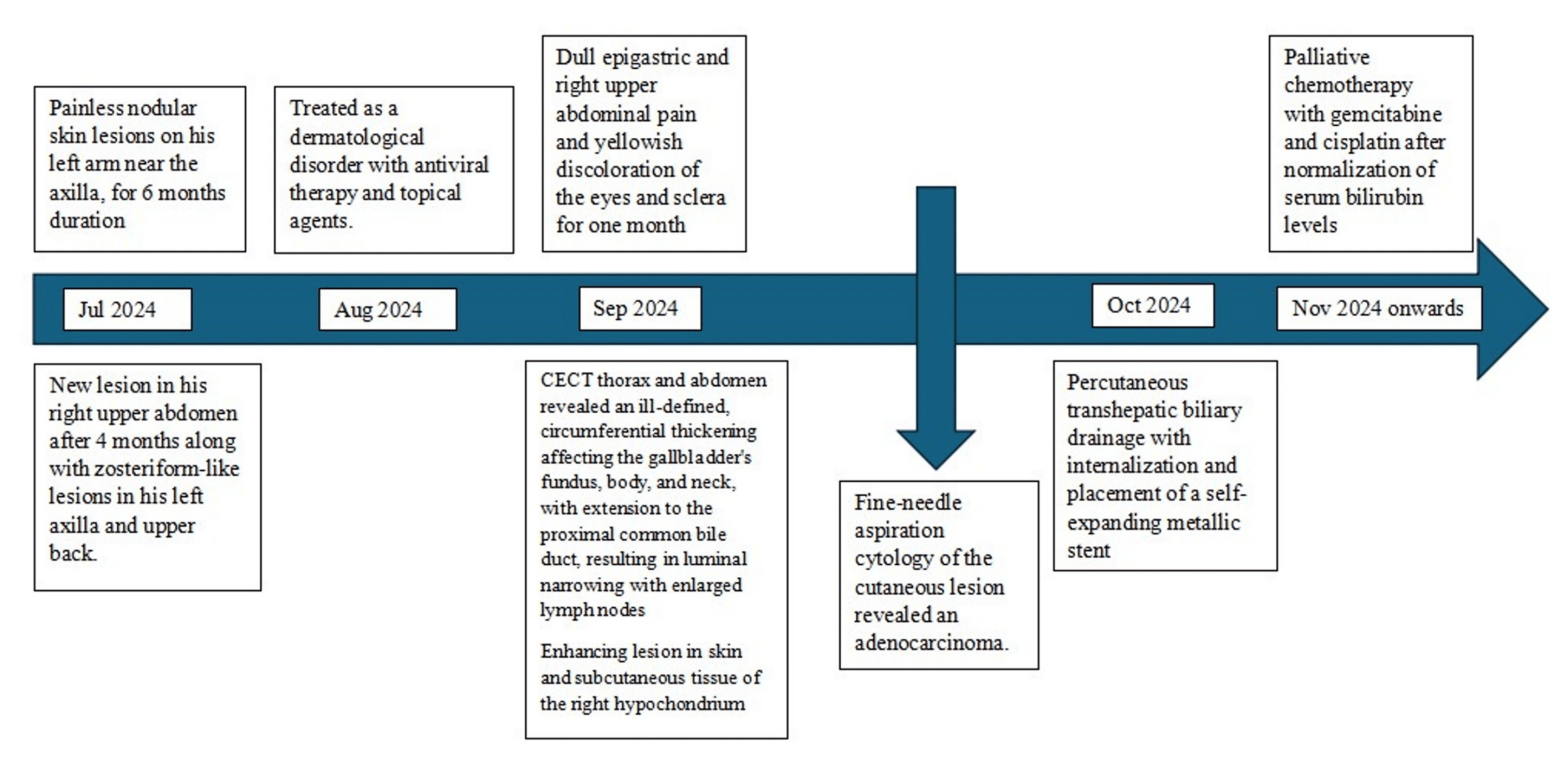
Timeline of events. CECT: contrast-enhanced computed tomography

Based on clinical, radiological, and pathological evaluations, the patient was diagnosed with metastatic gallbladder carcinoma, presenting with cutaneous metastases complicated by obstructive jaundice. Percutaneous transhepatic biliary drainage with internalization and placement of a self-expanding metallic stent was performed. Palliative chemotherapy with gemcitabine and cisplatin was planned after normalization of serum bilirubin levels. A timeline of events is depicted in Figure 4.

## Discussion

Cutaneous metastases from internal malignancies manifest in three scenarios as follows: patients with a known malignancy developing cutaneous metastases; patients presenting with cutaneous metastases along with obvious malignancy, as in our case; and individuals presenting with cutaneous metastases without obvious malignancy.

In a comprehensive retrospective analysis at the Veterans Health Administration (USA), only 77 cases (0.07%) out of 100,453 cancer cases reviewed were reported to have cutaneous metastasis [5]. This rarity often leads to misdiagnosis of skin lesions, especially when they precede the diagnosis of malignancy. Clinically, cutaneous metastases present with diverse manifestations, ranging from dermal papules, subcutaneous nodules, and inflammatory patches to ulcerated lesions, bullous lesions, alopecia neoplastica, and zosteriform dermatosis. This broad spectrum underscores the challenge in their clinical recognition and highlights the need for vigilant assessment [2,6,7].

In the present case, cutaneous metastases were the initial presentation, exhibiting diverse manifestations, including painless nodular and zosteriform-like lesions in the same patient. This led to initial mismanagement, presumed as a dermatological disorder. For patients with a known or radiologically evident malignancy, fine-needle aspiration cytology is sufficient to diagnose cutaneous metastases. Fine needle aspiration cytology (FNAC), being minimally invasive, facilitates the prompt detection and management of cases where skin nodules are not clinically suspected to be malignant. However, when the primary tumor is known, cutaneous metastases indicate a poor prognosis. In cases where the primary tumor site is unknown, cytomorphology along with clinical and radiological findings can help suggest the likely site of origin. However, a biopsy followed by immunohistochemical analysis is required to confirm the diagnosis. The role of immunohistochemistry is crucial in confirming the primary site, as the cytomorphology of cutaneous metastases may be non-specific [8].

Cutaneous metastases signify a grave prognosis, often suggesting widespread disease progression or, rarely, primary tumor recurrence. Patients with skin metastases typically have a short survival time, averaging a few months, underscoring the critical need for prompt diagnosis through heightened vigilance and judicious use of investigations to improve outcomes [7].

## Conclusions

The rarity of cutaneous metastases in gallbladder cancer often leads to misdiagnosis and delayed treatment. The diverse clinical presentations of these cutaneous metastases and the limited awareness among clinicians can pose significant diagnostic challenges. Maintaining a heightened level of suspicion is crucial for prompt diagnosis. Early identification enables timely intervention, which can potentially enhance prognosis by addressing the systemic spread of cancer in its initial stages.
